# Protein Kinase CK2 Inhibition Down Modulates the NF-κB and STAT3 Survival Pathways, Enhances the Cellular Proteotoxic Stress and Synergistically Boosts the Cytotoxic Effect of Bortezomib on Multiple Myeloma and Mantle Cell Lymphoma Cells

**DOI:** 10.1371/journal.pone.0075280

**Published:** 2013-09-27

**Authors:** Sabrina Manni, Alessandra Brancalion, Elisa Mandato, Laura Quotti Tubi, Anna Colpo, Marco Pizzi, Rocco Cappellesso, Fortunato Zaffino, Speranza Antonia Di Maggio, Anna Cabrelle, Filippo Marino, Renato Zambello, Livio Trentin, Fausto Adami, Carmela Gurrieri, Gianpietro Semenzato, Francesco Piazza

**Affiliations:** 1 Department of Medicine, Hematology and Clinical Immunology Branch, University of Padova, Padova, Italy; 2 Myeloma and Lymphoma Pathobiology Laboratory, Hematologic Malignancies Unit, Venetian Institute of Molecular Medicine, Padova, Italy; 3 Department of Medicine, General Pathology and Cytopathology Unit, University of Padova, Padova, Italy; University of North Carolina at Chapel Hill, United States of America

## Abstract

CK2 is a pivotal pro-survival protein kinase in multiple myeloma that may likely impinge on bortezomib-regulated cellular pathways. In the present study, we investigated CK2 expression in multiple myeloma and mantle cell lymphoma, two bortezomib-responsive B cell tumors, as well as its involvement in bortezomib-induced cytotoxicity and signaling cascades potentially mediating bortezomib resistance. In both tumors, CK2 expression correlated with that of its activated targets NF-κB and STAT3 transcription factors. Bortezomib-induced proliferation arrest and apoptosis were significantly amplified by the simultaneous inhibition of CK2 with two inhibitors (CX-4945 and K27) in multiple myeloma and mantle cell lymphoma cell lines, in a model of multiple myeloma bone marrow microenvironment and in cells isolated from patients. CK2 inhibition empowered bortezomib-triggered mitochondrial-dependent cell death. Phosphorylation of NF-κB p65 on Ser529 (a CK2 target site) and rise of the levels of the endoplasmic reticulum stress kinase/endoribonuclease Ire1α were markedly reduced upon CK2 inhibition, as were STAT3 phospho Ser727 levels. On the contrary, CK2 inhibition increased phospho Ser51 eIF2α levels and enhanced the bortezomib-dependent accumulation of poly-ubiquitylated proteins and of the proteotoxic stress-associated chaperone Hsp70. Our data suggest that CK2 over expression in multiple myeloma and mantle cell lymphoma cells might sustain survival signaling cascades and can antagonize bortezomib-induced apoptosis at different levels. CK2 inhibitors could be useful in bortezomib-based combination therapies.

## Introduction

Bortezomib, a boronic acid compound targeting the chymotrypsin-like activity of the 26S subunit of the proteasome, is a first-in class proteasome inhibitor (PI) [1], which has demonstrated remarkable activity against multiple myeloma (MM) and mantle cell lymphoma (MCL), two yet incurable hematologic malignancies [2], [3], [4]. At present, bortezomib-based combination therapies, incorporating both traditional chemotherapeutic drugs and novel agents, represent the standard care in MM and in MCL non Hodgkin Lymphomas [5], [6], [7], [8].

The mechanisms of bortezomib-induced apoptosis are only partially known. Initial findings described that it can affect the activation of the canonical NF-κB pathway because of the induced stabilization of IκBα, the physiological NF-κB inhibitor [9]. However, recent studies have demonstrated that bortezomib can also trigger NF-κB activity in MM cells [10]. However, bortezomib may also induce many other effects. For instance, it stabilizes the tumor suppressor p53 and the pro-apoptotic protein Bax and up regulates the proteins Noxa and Puma [11], while it induces cleavage and inactivation of the anti-apoptotic molecule Mcl1 [12], [13], thereby causing the activation of the mitochondria-dependent apoptosis. Bortezomib can also induce endoplasmic reticulum (ER) stress, which is a mechanism of critical importance for MM plasma cell survival due to chronic ER loading with a burden of perpetually synthesized immunoglobulins [14], [15]. A terminal pro-apoptotic unfolded protein response (UPR) is elicited [16] as a result of bortezomib treatment.

CK2 is a multifaceted Serine/Threonine kinase involved in several cellular processes and over-expressed and over-active in many solid and blood tumors [17], [18]. A number of studies have shown that CK2 over-expression may force the cell to acquire a pro-survival program through the direct or indirect regulation of critical molecules or signaling cascades [17], [19]. Interestingly, CK2 plays a central role in the activation of many cellular protein kinases by direct regulation of the activity of the chaperone complex formed by the molecules Cdc37 and Hsp90 [20], [21]. CK2 also regulates signaling cascades and molecules that are targeted by bortezomib. For instance, CK2 modulates IκBα protein turnover [22], [23], p53 function [24], [25], AKT activation [26] and the ER stress/UPR [27], [28], [29], [30]. We previously described that CK2 supports MM cell survival and its inhibition enhances the cytotoxic effect of both conventional chemotherapeutic agents such as melphalan [31], as well as of novel agents targeting Hsp90, both *in vitro* and *in vivo* [29]. Interestingly, CK2 was found to impinge on the proper activation of NF-κB and STAT3 in MM cells [31]. Most importantly, a phase I clinical trial with the oral ATP-competitive CK2 inhibitor CX-4945 (Cylene Pharmaceuticals, USA) is currently ongoing in the USA on relapsed/refractory MM patients [32], [33].

With the above as background, we hypothesized that CK2 could modulate the sensitivity of malignant cells to proteasome inhibitors. In the present work, we studied the effect of CK2 inhibition on bortezomib-induced cytotoxicity and evaluated the signaling pathways potentially counteracting bortezomib action in MM and MCL patients. We demonstrated that CK2 inhibitors cooperate with bortezomib in causing MM and MCL cell apoptosis by down modulating the signalling cascades of NF-κB and STAT3 and by potentiating the proteotoxic effects due to proteasome blockage. The data offer the rationale for the use of CK2 inhibitors in bortezomib-based combination therapies in these malignancies.

## Methods

### Ethics statement

Samples and biopsies from normal bone marrows**,** 26 MCL, 5 monoclonal gammopathy of undetermined significance (MGUS) and 20 MM patients' were processed and used after achievement of written informed consent according to the declaration of Helsinki. The project outline and consent procedures and forms were submitted and approved by the Ethic Committee of the Padova University Hospital (protocol number 2612P).

### Patients and cell cultures

Malignant CD138^+^ plasma cells were purified using the Rosette Sep Kit according to the Manufacturer's protocol (Stem cell Technologies, USA). Normal peripheral blood mononuclear cells (PBMC), MM cell lines RPMI 8226, U-266 and INA-6, the human stromal cell line HS-5 were isolated and cultured as described in [29], [34]. PBMC from MCL patients were obtained from peripheral blood or bone marrow as per standard Ficoll Paque® protocol. MM International Staging System (ISS) [35] and Mantle Cell Lymphoma International Prognostic Index (MIPI) [36] were applied to the patients population analyzed. MCL cell lines Granta-519, Jeko-1 and Rec-1 purchased from Deutsche Sammlung von Mikroorganismen und Zellkulturen (DSMZ, Germany) were cultured in DMEM supplemented with 10% fetal bovine serum (FBS), RPMI 1640 supplemented with 20% FBS, and RPMI supplemented with 10% FBS, respectively. Testing for Mycoplasma infection was carried at a monthly basis.

### Cytokines and chemicals

Interleukin-6 (IL-6) was purchased from Sigma-Aldrich, Italy. K27 (2-amino-4,5,6,7-tetrabromo-1*H*-benzimidazole) was synthesized and kindly provided by Dr Z. Kazimierczuk (Warsaw University, Poland). CX-4945 was from Activate-scientific, Germany. The specificity and mechanism of action of these CK2 inhibitors were previously characterized [29], [32], [39]. Bortezomib was purchased from Selleck chemicals, USA.

### Histological and immunohistochemical analysis

Immunohistochemistry was performed on 4–5 µm-thick formalin-fixed and paraffin-embedded (FFPE) sections from each tumor sample with the following primary antibodies, according to the manufacturer’s instructions: CK2α (rabbit, clone EP1963Y, Epitomics, Burlingame, CA, USA; working dilution 1∶50, 60 min, EDTA buffer), CK2β (mouse, clone 6D5, Santa Cruz Biotechnology, Santa Cruz, CA, USA; working dilution 1∶250, 30 min, EDTA buffer), p-STAT3 Ser727 (rabbit, polyclonal, SAB Signalway Antibody, Pearland, TX, USA; working dilution 1∶200, 30 min, citrate buffer); Cyclin D1 (Dako, clone EP12, working diluition 1∶20); CD138 (Dako, clone M15). All sections were processed using the sensitive Bond Polymer Refine Detection kit, a biotin-free, polymeric horseradish peroxidase–linker antibody conjugate system, in an automated immunostainer (Bond maX, Menarini, Florence, Italy), as described elsewhere [37], [38]. Sections were then slightly counterstained with hematoxylin. Appropriate positive and negative controls were run concurrently. Nuclear immunolabeling was semiquantitatively scored in a four-tier scale (0 = 0–5%, 1 = 6–33%, 2 = 34–66%, and 3 = 67–100% positive cancer cells). Nuclei of neoplastic cells within each section were also graded for intensity (0 = absent, 1 = weak, 2 = moderate, and 3 = strong immunoreaction). Specimens were scored independently by three pathologists (FM, MP, and RC) and a consensus was reached.

### Assessment of drug concentration-effect and calculation of the combination index (CI)

The experiments were performed as described in [29].

### mRNA silencing

RNA interference was performed using the Amaxa® system (Cell Line Nucleofector® Kit V, L-022 program, Lonza, Italy), with siGLO Green scrambled siRNAs, CK2α and CK2β targeting siRNAs (100 pmoles each) (Thermo Scientific). CK2α target sequence were: GCAUUUAGGUGGAGACUUC; GGAAGUGUGUCUUAGUUAC; GCUGGUCGCUUACAUCACU; AACAUUGUCUGUACAGGUU. CK2β target sequences were: CAACCAGAGUGACCUGAUU; GCAAGGAGACUUUGGUUAC; GCAAUGAAUUCUUCUGUGA; CCAAGUGCAUGGAUGUGUA.

### Evaluation of growth and apoptosis

Apoptosis was assessed by Annexin V/Propidium Iodide staining (BD Pharmingen) or, in separate experiments, by detection of mitochondrial membrane potential using the 5,5′,6,6′, tetrachloro-1,1’,3,3’-tetraethylbenzimidazolyl carbocyanin iodide dye (JC-1) (Trevigen, Germany) according to the manufacturers’ instructions. Cells were labeled with Cy5- or FITC-conjugated annexin (Becton-Dickinson, Italy). APC-conjugated anti-CD45, APC-conjugated anti-CD19 and PE Cy5-conjugated anti-CD38 were from Becton-Dickinson, Italy. Fluorescence Activated Cell Sorting (FACS) analysis was performed using a FACS-Calibur Cell Cytometer and the CellQuest® software (Becton-Dickinson, Italy)

### Determination of ATP cell content

ATP production was measured using the ATP Lite 1 step system (Perkin Elmer, Italy).

### Western blot (WB)

WB was performed according to standard protocols. Antibodies used were: CK2α [31]; PARP, IRE1α, p-STAT3 Ser 727, total STAT3, Bcl2, Mcl1, p-eIF2α Ser 51, total eIF2α (Cell signaling Technology, MA, USA); Hsp70, total p65-NF-κB, phospho-Ser 13 Cdc37 (Abcam, UK); p-NF-κB p65 Ser 529, total Cdc37 (Santa-Cruz Biotechnology, CA, USA), GAPDH (Ambion, USA); β-actin (Sigma-Aldrich, Italy); Bak (Merck, MA, USA); Bax, CK2β (Becton Dickinson, Italy); poly-Ubiquitin (Enzo Life Sciences, Italy). At least n = 2 experiments for each WB were performed.

### Real time polymerase chain reaction

Performed as described in [29]. The primers used for *CYCLIN D1* were: Forward 5′-GCAAATGGAGCTGCTCCTG-3′ and Reverse 5′-GCGTGTTTGCGGATGATCTG-3′, for *IAP-2* Forward 5′-GACAGGAGTTCATCCGTCAAG-3′ and Reverse 5′-TTCCACGGCAGCATTAAT-3′ and for *β-ACTIN*: Forward 5′-CAGCTCACCATGGATGATG-3′ and Reverse 5′-ATGCCGGAGCCGTTGTC-3. Real time PCR for *TNFα, COX-2, IL-6, Bcl2, NOS-2* was performed using the “Real time PCR assay for monitoring NF-κB-regulated genes” from Signosis (USA).

### Statistical analysis

Data obtained were evaluated for their statistical significance with the two-tail paired Student’s *t* test or analysis of variance (ANOVA) with post-hoc corrections. Values were considered statistically significant at *p* values below 0.05.

## Results

### CK2 is highly expressed in MM and MCL, two bortezomib sensitive blood tumors and is essential for MCL cell survival

**Figure 1:**
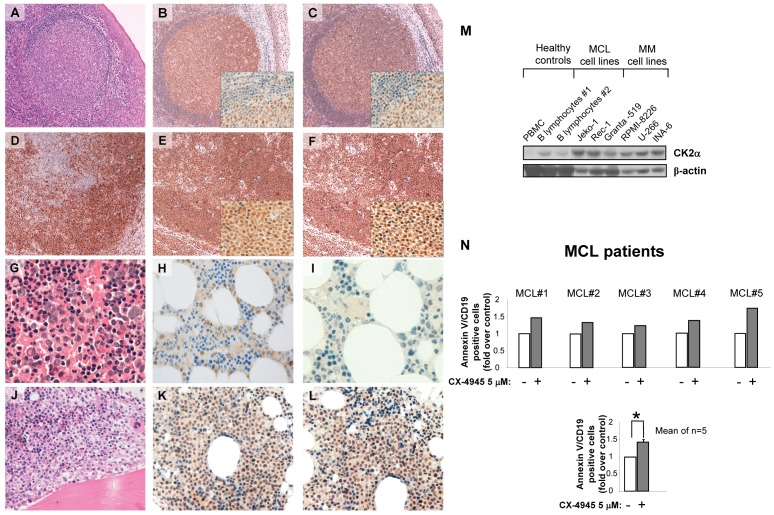
CK2 expression in MCL and MM and effects of CK2 inhibition on MCL cell survival. (A-L). Representative immunohistochemistry of CK2α CK2β in normal lymphoid tissue, MCL, MGUS and MM patient specimens. H&E (A), CK2α(B) and CK2β (C) staining of normal reactive lymphoid tissue. Staining for Cyclin D1 (D), CK2α (E) and CK2β (F) in MCL lymph node. Bone marrow biopsy from MGUS stained for H&E (G), CK2α (H) and CK2β (I); MM bone marrow biopsy stained for H&E (J), CK2α (K) and CK2β (L). (H&E and immunoperoxidase stain; original magnification: 10x and 20x; pictures B, C, E, F have an insert at 100x magnification to show details). (M) WB analysis of CK2α expression. From left to right: 1 normal PBMC, 2 normal B lymphocytes (B Lymph), 3 MCL cell lines, and 3 MM cell lines. β-actin was used as a loading control. (N) Quantification of apoptosis through annexin V staining and FACS analysis in CD19^+^ B lymphocytes from five MCL patients treated with the CK2 inhibitor CX-4945 (5 µM). Data represent results of the five separate patients sample (upper panel) and the mean ± SEM of five independent experiments. * indicates p<0.05.

To investigate the expression of CK2 in bortezomib-sensitive B cell tumors, the expression of CK2α (catalytic subunit) and CK2β (regulatory subunit) proteins were examined by immunohistochemistry in lymph node biopsies from MCL patients (n = 21), in normal lymphoid tissues (tonsils or reactive lymph nodes)(n = 3), in bone marrow biopsies obtained from monoclonal gammopathy of undetermined significance (MGUS) (n = 5) and MM patients (n = 17). As shown in Figure 1, panels B and C, CK2α and CK2β were found mostly expressed in the germinal centers (GC) of normal reactive lymph nodes (hematoxylin and eosin (H&E) staining in Figure 1A), while a much lower expression of both CK2 subunits was detected in the mantle zone. Conversely, the paracortical zone, in which mostly T-cells reside, showed strong and diffuse nuclear positivity for CK2. Remarkably, in lymph nodes of 62% of MCL patients in our series (13/21 cases) both CK2α and CK2β were strongly expressed in tumor cells as compared to normal residual areas and their expression overlapped the expression pattern of Cyclin D1 (Figure 1, panels D-E-F). In the BM of MGUS patients CK2α and CK2β expression was scattered in hematopoietic cells of myeloid and erythroid lineage as well as in megakaryocytes but plasma cells did not display a significant over expression of the kinase as compared to normal hematopoietic cells (Figure 1H-I; figure 1G shows H&E staining). On the opposite, in 88% of MM patients analyzed (15/17 cases) CK2 was found highly expressed by malignant plasma cells in the BM (Figure 1, panels K-L; Figure 1J shows H&E staining). In the case of MGUS and MM samples, double immunostaining for CD138 and either CK2α or CK2β is also shown in supplemental Figure S1 to emphasize the expression of the kinase in non malignant (Figure S1 A, B), pre-malignant MGUS (Figure S1 C, D) and malignant MM (Figure S1 E, F) bone marrow plasma cells. According to our findings, MCL and MM cases were divided in two groups (CK2 high and CK2 low) based on the intensity of CK2 expression. The features of MCL, MGUS and MM patients analyzed, the description of the scoring method used to estimate CK2 subunits intensity of expression and the respective results are summarized in Tables 1 and 2 and 3. Eighty percent of the CD138+ MGUS plasma cells displayed a low staining for CK2α/CK2β, while CD138+ MM plasma cells displayed high scores for CK2α (88%) and CK2β (64%).

**Table 1:** Clinical and pathological features of MCL cases analyzed.

Sample	Age/Sex	Stage	MIPI	IMMUNOHISTOCHEMISTRY									IgH/CCND1 FISH	Karyotype	alive = A death = D at 3 years
				P-STAT3	CK2α	CK2β	CD5	CD19	CD20	CD23	CYCLIN D1	%Ki-67 positive cells			
**CK2 high**															
MCL01	71/F	IVA	5.8	2	1	2	+	+	+	+	+	ND	NV	NV	A
MCL02	52/M	IVA	5.3	2	2	3	+	+	+	-	+	ND	+	t(14;11)	A
MCL03	55/M	IVA	5.2	3	2	2	.	ND	+	-	+	20	Normal	Normal	A
MCL04	83/F	IVB	6.4	2	1	2	+	ND	+	-	+	40	-	Normal	A
MCL05	76/M	IVA	6.6	3	2	3	+	ND	+	-	+	30	NA	NA	A
MCL06	60/M	IVA	5.7	2	2	3	+	+	+	-	+	ND	NA	NA	A
MCL07	80/M	IVA	6.1	1	3	1	-	+	+	-	-	ND	NA	NA	D
MCL08	67/M	IVB	6	2	2	2	+	+	+	-	+	15	-	Normal	A
MCL09	65/M	IVA	5.4	1	2	2	+	+	+	-	-	ND	ND	ND	A
MCL10	64/M	IVA	4	2	2	3	+	-	+	-	-	ND	+	NA	A
MCL11	51/F	IIA	5.9	2	2	3	-	ND	+	-	+	41	ND	Normal	A
MCL12	34/M	IIIA	ND	2	2	2	+	+	+	-	+	ND	-	Normal	A
MCL13	74/F	IVA	6.1	2	2	2	+	+	+	-	+	ND	ND	ND	A
**CK2 low**															
MCL14	76/M	IIIA	6.7	2	1	1	+	ND	+	-	+	ND	Normal	Normal	A
MCL15	53/M	IVB	6.7	2	1	1	+	+	+	-	+	50	+	Normal	A
MCL16	70/M	IVA	6.2	3	0	0	+	+	+	-	-	10	-	NA	A
MCL17	71/F	IVA	5.9	1	1	1	+	+	+	-	+	ND	+	NA	A
MCL18	74/F	IVA	6.3	1	1	1	-	+	+	-	+	ND	+	Hyperdiploid	A
MCL19	72/F	IVB	7.3	0	0	0	+	+	+	-	+	ND	-	Hyperdiploid	D
MCL20	65/F	IVA	5.8	3	1	1	+	+	+	+	+	20	NA	NA	A
MCL21	57/F	IVA	7.1	2	1	1	+	+	+	-	+	50	+	Hypodiploid	A

Cases were progressively numbered and sorted in high and low CK2 expressing subgroups. Scoring method used was: 0 = 0–5% positive cells; 1 = 6–33% positive cells; 2 = 34–66% positive cells; 3 = 67–100% positive cells. Cases were defined as CK2 high if the sum of the scores for CK2α and CK2β was equal to or > 3 and CK2 low if the sum was < 3; MIPI = mantle cell international prognostic index according to (44). M = male; F = female; FISH =  fluorescence in situ hybridization; ND = not determined; NA = not assessable; P-STAT3  =  Ser 727 phosphorylated STAT3.

**Table 2:** Clinical and pathological features of MGUS and MM cases analyzed.

Sample	Age/sex	Paraprotein type	Durie-Salmon	ISS	% bone marrow PC	Cytogenetics	FISH	P-STAT3	CK2α	CK2β	alive = A death = D after 3 years
**MGUS**											
MG1	49/M	IgG/κ	-	-	10	Normal	NA	1	0	0	A
MG2	68/F	IgG/κ	-	-	10	Normal	NA	1	1	1	A
MG3	57/M	IgM/κ	-	-	3	NA	NA	2	1	1	A
MG4	40/F	IgG/λ	-	-	10	NA	NA	1	1	1	A
MG5	78/M	IgG/κ	-	-	5	Normal	NA	2	2	2	A
**MM**											
**CK2 high**											
MM01	72/M	κ	IIA	II	90	ND	ND	3	3	3	D
MM02	58/F	λ	IIIB	II	90	Normal	ND	-	3	-	A
MM03	66/F	IgA/λ	IIIA	II	90	Hyperploid	Normal	3	3	2	A
MM04	69/M	κ	IIA	III	70	ND	ND	0	3	2	D
MM05	42/F	IgG/κ	IIA	III	70	Hyperploid	13q14	3	3	2	D
MM06	72/M	IgA/κ	IIIA	III	100	t(11;14); monosomy 8; rearrang 1	ND	2	3	3	D
MM07	83/F	IgA/κ	IIIA	II	90	Normal	Normal	3	3	3	A
MM08	76/M	IgG/λ	IIIA	II	62	Hyperdiploid	NA	3	2	2	A
MM09	69/M	κ	IIA	III	70	Hypodiploid	biallelic del(13q14)	2	2	2	A
MM10	69/M	IgG/λ	IIA	II	70	t(11;14)	t(11;14)	2	2	2	A
MM11	78/M	IgG/κ	IIA	II	70	Hyperdiploid	IGH	3	3	3	A
MM12	66/F	IgA/λ	IIA	III	70	ND	ND	3	3	3	D
MM13	75/M	IgG/κ	IIA	III	57	Hyperdiploid	biallelic del(13q14)	2	3	-	D
MM14	73/M	IgA/κ	IIIA	II	60	Hyperdiploid	13q14	2	2	1	A
MM15	75/M	κ	IIB	III	60	Hyperdiploid	Chr.11 trisomy; rearranged Chr14	0	2	1	D
**CK2 low**											
MM16	63/F	IgD/λ + λ	IIIA	III	100	NA	t(11;14)	3	1	0	A
MM17	69/F	IgG/λ	IIIA	III	90	Hypodiploid	13q14	1	-	1	A

Cases were progressively numbered and sorted in high and low CK2 expressing subgroups. Scoring method used was: 0 = 0–5% positive cells; 1 = 6–33% positive cells; 2 = 34–66% positive cells; 3 = 67–100% positive cells. Cases were defined as CK2 high if the sum of the scores for CK2α and CK2β was equal to or > 3 and CK2 low if the sum was < 3. Clinical staging was performed according to Durie-Salmon criteria and according to the International Staging System (ISS; albumin levels ≤ or > 35 g/L; β2microglobulin levels ≤ or > 3.5 mg/L) (43). M = male; F = female; PC = plasma cells; FISH =  fluorescence in situ hybridization; ND = not determined; NA = not assessable.

**Table 3:** Summary of CK2α, CK2β and STAT3 positivity scores in CD138+ plasma cells of MGUS and Multiple Myeloma cases.

	MGUS (n = 5)				Multiple Myeloma (n = 17)			
Score*	0	1	2	3	0/NA	1	2	3
**CK2α n (%)**	1 (20%)	3 (60%)	1 (20%)	0 -	1 (6%)	1 (6%)	5 (29%)	10 (59%)
**CK2β n (%)**	1 (20%)	3 (60%)	1 (20%)	0 -	3 (18%)	3 (18%)	6 (35%)	5 (29%)
**P-STAT3 n (%)**	0 -	3 (60%)	2 (40%)	0 -	3 (18%)	1 (6%)	5 (29%)	8 (47%)

* Nuclear immunolabeling semi quantitatively scored in a four-tier scale: 0 = 0–5%, 1 = 6–33%, 2 = 34–66%, and 3 = 67–100% positive CD138+ cancer cells.

NA = not assessed.

Furthermore, by western blot analysis we found that CK2α was expressed at relatively low levels in normal B lymphocytes, while in all the MCL and MM cell lines tested it was found highly expressed (Figure 1M). To note, the expression of CK2α was found to be higher in the Rec-1 MCL cell line, which is fairly less sensitive to bortezomib [40]. The features of the MCL and MM cell lines used are listed in supplemental Table 4 and 5.

**Table 4:** Characteristics of MCL cell lines used in the study.

Cell line	Origin	Disease status	CYCLIN D1 expression	p53 status	Karyotype
Granta-519	PB	Relapsed leukemic MCL	+	wt	Hypodiploid with 8% polyploidy*
Jeko-1	PB	Leukemic conversion of MCL, large variant	+	del/mut	Hypertriploid°
Rec-1	LN or PB	DLBCL progressing to trasformed MCL, blastoid variant	+	wt	Near-diploid, with 25% polyploidŷ

* t(11;14) and rearrangement at 9p22 associated with CCND1 (Cyclin D1) activation and deletion of p15/p16;

° Cryptic rearrangements of CCND1 and IgH;

? t(11;14).

Abbreviations: PB: peripheral blood; MCL: mantle cell lymphoma; LN: lymph node, DLBCL: diffuse large B cell lymphoma; wt: wild-type; del/mut: deleted/mutated.

**Table 5:** Characteristics of MM cell lines used in the study.

Cell line	Origin	Paraprotein type	Karyotype
U-266	PB	IgE secreting MM (refractory-terminal)	Hypodiploid with 6.5% polyploidy
RPMI-8226	PB	IgG (only λ light chain) secreting MM	Hypotriploid with 7.5% polyploidy
INA-6	Pleural Effusion	IgG/κ chain secreting plasma cell leukemia	Tetraploid with 10-25% polyploidy

Abbreviations: PB: peripheral blood; MM: multiple myeloma.

### CK2 inhibitors leads to MCL cell apoptosis

Next, in order to see whether - alike to MM cells - CK2 could control MCL survival, we assessed the effects of the clinically tested ATP-competitive CK2 inhibitor CX-4945 on MCL cells isolated from five MCL patients. As shown in Figure 1N, CX-4945 caused an increase of apoptotic annexin V-expressing CD19^+^ MCL B cells.

Altogether, these data indicate that protein kinase CK2 is over expressed in a substantial fraction of MCL and MM, both in tissue biopsies and in cell lines, and that MCL is another B-cell malignancy, whose growth could be regulated by CK2.

### Inhibition of CK2 in MM and MCL cells empowers bortezomib-induced cell proliferation in a synergistic mode

**Figure 2:**
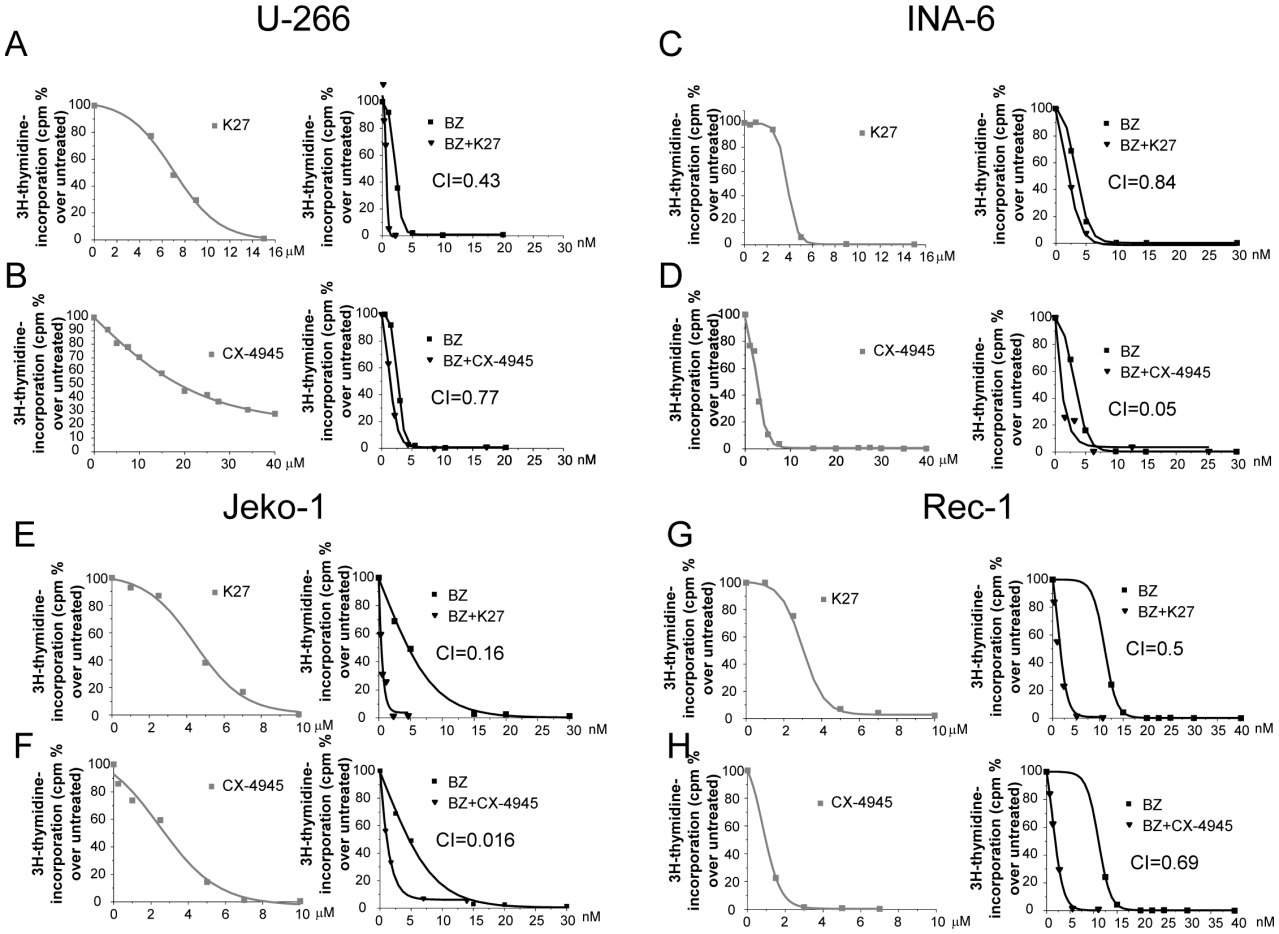
Effects of CK2 and proteasome inhibition on MM and MCL cell proliferation. Synergistic effect of K27 and bortezomib (BZ in the figure) (A, C, E, G) or CX-4945 and bortezomib (B, D, F, H) on U-266 (A, B), INA-6 (C, D), Jeko-1 (E, F) and Rec-1 (G, H) cell proliferation. Left graphs: dose-response curve of cells incubated for 48 hours with increasing concentrations of K27 or CX-4945 (grey squared curve). Right graphs: dose-response curve of cells incubated for 48 hours with increasing concentrations of bortezomib alone (black squared curve) or K27 or CX-4945 plus bortezomib (black triangle curve). Cell proliferation was assessed by ^3^H-thymidine-incorporation assay. IC_50_ for K27 was 6.98 µM and for bortezomib 2.16 nM in U-266; 3.77 µM for K27 and 3.18 nM for bortezomib in INA-6 cells; 4.43 µM for K27 and 4.65 nM for bortezomib in Jeko-1 cells and 3.13 µM for K27 and 11.06 nM in Rec-1 cells. IC50 of CX-4945 was 19.8 µM in U-266, 2.42 µM in INA-6, 2.4 µM in Jeko-1 and 1.46 µM in Rec-1 cells. The CI between K27 and bortezomib was calculated as to be 0.43 for U-266, 0.84 for INA-6, 0.16 for Jeko-1 and 0.5 for Rec-1. The CI between CX-4945 and bortezomib was calculated as to be 0.77 for U-266, 0.05 for INA-6, 0.016 for Jeko-1 and 0.69 for Rec-1.

Then, we investigated whether CK2 inhibition could affect the cytotoxic effects of bortezomib on MM and MCL cell lines. Employing CX-4945 and the previously described tTBB-derivative K27 [41], a first set of experiments tested whether the simultaneous inhibition of CK2 and the proteasome could cause either an additive or a synergic effect in terms of cell growth arrest. To this aim, we performed ^3^H-thymidine incorporation assays evaluating the rate of cell proliferation at increasing concentration of bortezomib (range: 1–30 nM), K27 (range: 1–15 µM) and CX-4945 (range: 1–40 µM) and the combination of bortezomib either with K27 or CX-4945. The results were analyzed to obtain the IC_50_ for the three agents and the *constant ratio drug combination assay* was performed, giving the combination indexes (CI) according to the method described in [29]. The results showed that treatment of MM cells (Figure 2A-D) or MCL cells (Figure 2E-H) with bortezomib and CK2 inhibitors was strongly synergic, as judged by the CI well below 1 (ranging from 0.05 to 0.84 for MM cell lines and from 0.016 to 0.69 for MCL cell lines) in all the conditions tested, and in the case of MCL cells, independently of the degree of sensitivity to bortezomib.

### CK2 inhibitors enhance bortezomib-triggered MM and MCL cell apoptosis

**Figure 3:**
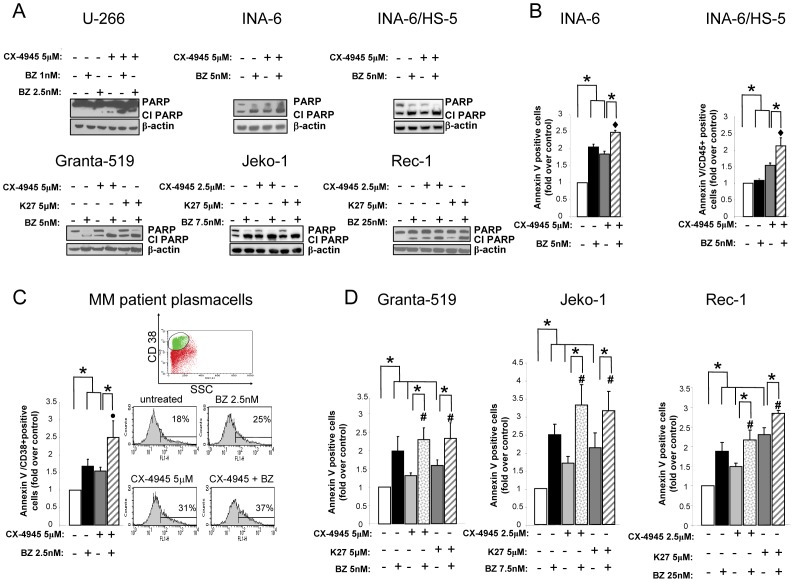
Effects of CK2 and proteasome inhibition on MM and MCL cell survival. Evaluation of apoptosis through WB analysis of PARP cleavage (CL PARP =  Cleaved PARP) (A) in MM cells U-266, INA-6, INA-6 co-cultured with HS-5 (top panels), MCL cells Granta-519, Jeko-1 and Rec-1 (bottom panels); (B, C) annexin V staining and FACS analysis on INA-6 MM cells alone or in co-cultures with HS-5 (B), patient derived plasma cells (C) treated with the CK2 inhibitors CX-4945 (grey bars), the proteasome inhibitor bortezomib (BZ in the figure) at different concentrations (black bars) or the combination of the two compounds (grey striped bars) for 18 hours. In the case of INA-6 grown in co-colture with HS-5, experiments were performed by staining with APC-conjugated anti-CD45 antibody, which is expressed by INA-6 cells but not by stromal cells and with FITC-conjugated annexin V. In C, the apoptotic effect was measured by double staining with CD38 PE and annexin V-FITC. CD38^+^ bright cells were selected. Histogram bars represent mean ± SEM of three independent patient plasma cells (left panel), while the cytometric histograms show the percentage of annexin V positive cells in a representative experiment (right panel); (D) apoptosis of MCL cell lines Granta-519, Jeko-1, Rec-1 treated with the CK2 inhibitors CX-4945 (light grey bars), K27 (dark grey bars), the proteasome inhibitor bortezomib at different concentrations (black bars) or the combination of CX-4945 and bortezomib (grey dotted bars) or K27 and bortezomib (grey striped bars) for 18h. Data represent mean ± SEM of at least three independent experiments. * indicates p<0.05. In B ♦ indicates p<0.05 between samples treated with bortezomib 5 nM alone and bortezomib 5 nM together with CX-4945. In C • indicates p<0.05 between samples treated with bortezomib 2.5 nM alone and bortezomib 2.5 nM together with CX-4945. In D # indicates p<0.05 between samples treated with bortezomib alone and bortezomib together with CX-4945 or K27.

We next assessed the extent of apoptosis upon CK2 inhibitors and bortezomib. CX-4945 caused an increase of bortezomib-induced MM and MCL cell apoptosis, as assessed by western blot evaluation of PARP cleavage (Figure 3A). Annexin V staining and FACS analysis of MM cell line INA-6 grown either with IL-6 or in co-cultures with HS-5 bone marrow stromal cells (BMSC) (Figure 3B), freshly isolated MM plasma cells from patients (Figure 3C) and MCL cell lines (Figure 3D) also demonstrated a cooperation between CK2 inhibitors and bortezomib in inducing cell apoptosis (p<0.05, n = 3–6). The effectiveness of HS-5 bone marrow stromal cells to induce INA-6 cell resistance to chemotherapy was confirmed in experiments employing doxorubicin as a control (Figure S2 A). Moreover, similar results on the enhanced bortezomib cytotoxicity upon CK2 inhibition were obtained with the other CK2 inhibitor K27 (Figure S2 B). To note, the enhanced apoptotic effect of the combination of bortezomib and CK2 inhibitors was seen on all the MCL cell lines used, irrespective of their different sensitivity to bortezomib [40]. Significantly, the analysis of the effects of this treatment on normal B lymphocytes obtained from healthy donors showed that the exposure to bortezomib, K27, CX-4945 or the combination of the drugs induced a much lower rate of apoptosis, which was never cooperative, indicating little toxicity on normal cells (p>0.05, n = 5–7, Figure S2 C). Lastly, to further validate these findings, INA-6 and Rec-1 cells were subjected to the intracellular ATP generation assay, which measures cell proliferation and viability. As shown in Figure S2 D, both cell lines were greatly impaired in their capability to produce ATP upon treatment with bortezomib, K27, CX-4945. Most importantly, the combination of the two inhibitors with bortezomib caused a stronger reduction of ATP production (p<0.05, n = 3 for both cell lines).

Altogether, these data clearly indicate that CK2 inhibitors and bortezomib cooperate in inducing MCL and MM cell apoptosis.

### Combined treatment of CK2 inhibitors and bortezomib causes mitochondrial apoptosis of MM and MCL cells

**Figure 4:**
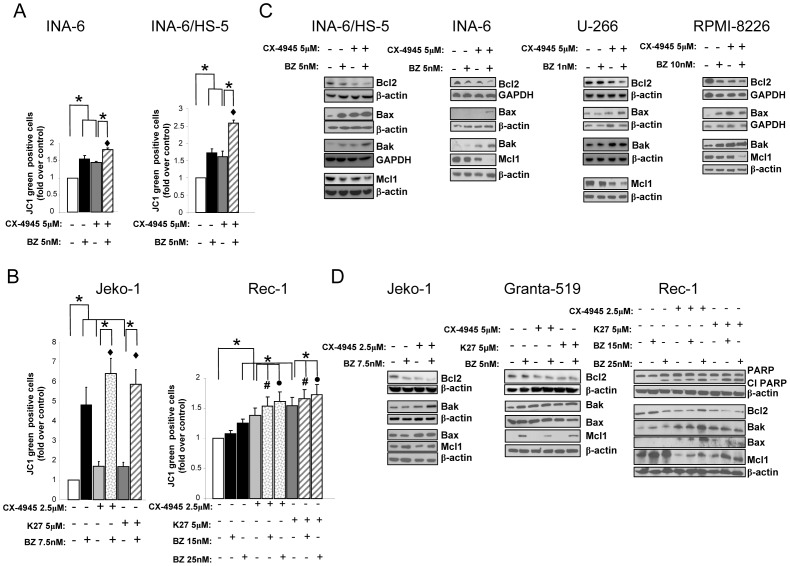
Combined treatment of CK2 inhibitors and bortezomib causes mitochondrial apoptosis of MM and MCL cells. JC1 staining and FACS analysis of MM cells (INA-6 or INA-6 cocoltured with HS-5 (A) or MCL cell lines (Jeko-1 or Rec-1)(B) treated with the CK2 inhibitors CX-4945 or K27, bortezomib (BZ in the figure) at different concentrations or the combination of CX-4945 or K27 and bortezomib for 18h. Data represent mean ± SEM of at least three independent experiments. * indicates p< 0.05. ♦ indicates p<0.05 between samples treated with bortezomib alone (at the dose indicated in figure) and bortezomib together with CX-4945. # indicates p<0.05 between samples treated with bortezomib 15 nM alone and bortezomib 15 nM together with K27 or CX-4945. • indicates p<0.05 between samples treated with bortezomib 25 nM alone and bortezomib 25 nM together with K27 or CX-4945. (C-D) WB analysis for expression of prosurvival signalling proteins (Bcl2, Mcl1) or pro-apoptotic proteins (Bax, Bak) in MM (C) or MCL (D) cell lines treated with the CK2 inhibitors CX-4945 or K27, bortezomib, or the combination of the two compounds for 18h. GAPDH or β-actin was used as a loading control.

To investigate the mechanisms leading MM and MCL cells to apoptosis upon exposure to CK2 inhibitors and bortezomib, we analyzed mitochondrial membrane potential by flow cytometry analysis of JC-1 dye [34] as well as Bcl2 family members expression by WB. As shown in Figure 4A and B, MM and MCL cell lines displayed an accumulation of JC-1 green upon the different treatments. The association of bortezomib with CK2 inhibitors caused a stronger mitochondrial membrane potential depolarization as compared to the single treatments (p<0.05, n = 3–6). Again, for MM cells this effect was present when cells were grown both in suspension and on bone marrow stromal cells HS-5 (which could confer drug resistance). For MCL cells, this effect was present both in Jeko-1 and in Rec-1 cells. Bcl2 family members expression analysis revealed that treatment with bortezomib plus CK2 inhibitors caused a stronger reduction of the anti-apoptotic Bcl2 and Mcl1 proteins and a higher accumulation of the pro-apoptotic Bak and Bax proteins in MM (Figure 4C) and MCL cells (Figure 4D). However, in MCL cells we observed a different pattern of changes since, at variance with MM cells and according to previous studies [40], bortezomib caused a rise of Mcl1 levels that was partly antagonized by the combination with CK2 inhibitors. In Granta-519 the most evident changes were for Bcl2 and Mcl1, while Bax and Bak seemed not to vary significantly. To note, in the case of Rec-1, a less bortezomib-sensitive cell line, apoptosis was achieved even by combining sub-apoptotic concentrations of bortezomib (15 nM) with CK2 inhibitors (as judged by JC-1 and PARP cleavage experiments and H3-thymidine incorporation, Figure 4B and 4D).

In sum, these results on changes in Bcl2 family proteins (even if somewhat variable among the various cell types) - taken together with all the other findings described above (H3-thymidine incorporation, PAPR cleavage analysis, annexin V staining, ATP generation assay, JC-1 staining) - clearly indicate that CK2 inhibitors and bortezomib strongly cooperate to destroy the mitochondrial homeostasis and the balance of Bcl2 family members, causing a shift towards the pro-apoptotic cascade.

### Bortezomib triggers CK2-dependent Cdc37 and NF-κB p65 phosphorylation

Since CK2 is a stress-responsive kinase [29], which protects from apoptosis, we asked whether it could be downstream from bortezomib-induced cellular stress. Thus, to check whether bortezomib affects CK2 activity, we treated MM and MCL cells with increasing concentrations of the proteasome inhibitor and assessed by western blot analysis the CK2-dependent phosphorylation of two known CK2 targets, i.e. the Hsp90 co-chaperone Cdc37 on Ser13 [20] and the NF-κB member p65 on Ser529 [42], [43]. As shown in Figure S3, bortezomib treatment for 8 hours at increasing concentrations (1, 5 or 10 nM) did not affect the high levels of Cdc37 Ser13 phosphorylation already present in MM cells; however, it caused a dose-dependent increase of NF-κB p65 Ser529 phosphorylation in all the three MM cell lines tested. On the opposite, in MCL cell lines Granta-519 and Jeko-1 we observed lower basal levels of phospho Ser13 Cdc37 but a dose-dependent increase upon bortezomib treatment; differently, in the MCL cell line Rec-1, the levels of phospho Ser13 Cdc37 were - like in MM cells - higher and not inducible. At variance with MM cell lines, phospho Ser529 p65 was inducible by bortezomib only in Granta-519, less in Jeko-1 but not at all in Rec-1 cells. Therefore, it is conceivable that this kinase is variably constitutive and active also on Cdc37 and NF-κB p65 in MM and MCL cells and can be increased by bortezomib.

### CK2 inhibition diminishes the constitutive STAT3 Ser727 phosphorylation and NF-κB activation in MM and MCL cells

**Figure 5:**
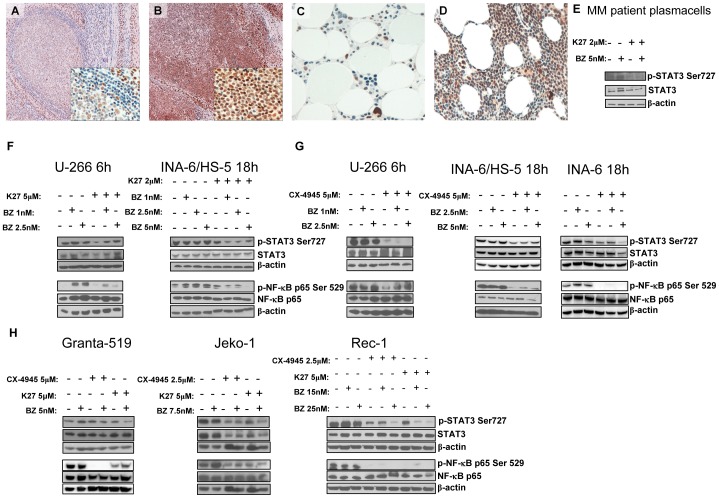
Immunohistochemical analysis of STAT3 phosphorylation on Ser727 in normal lymphoid tissue, MCL, MGUS and MM samples and effects of CK2 inhibition on STAT3 and NF-κB phosphorylation. (A-D) Immunohistochemistry for phosphorylated STAT3 on Ser727 in normal reactive lymphoid tissue (A), neoplastic mantle cells from MCL patient (B), MGUS (C) and MM patient (D) (Immunoperoxidase stain, original magnifications 20x; pictures A and B have an insert at 100x magnification to show details). (E-H) Expression of phosphorylated STAT3 on Ser727 (p-STAT3 Ser727), total STAT3, phosphorylated NF-κB on Ser529 (p-NF-κB p65 Ser529), total NF-κB, in MM patient derived plasma cells (E), MM cell lines (F, G), MCL cell lines (H) treated with K27 (or CX-4945) and bortezomib (BZ in the figure) at different doses and the combination of CK2 and proteasome inhibitors for 6h (F, G left panel) or 18h (F, G right panel and H). β-actin was used as a loading control.

CK2 activates survival pathways, which could be pivotal for the growth of both MM and MCL cells, namely the NF-κB and STAT3 cascades [44], [45], [46], [47], [48]. To check the STAT3 activation *status* in our patients' series, we analyzed the expression of phospho Ser727 STAT3 by immunohistochemistry in the same samples as in Figure 1. As shown in Figure 5A-D, accordingly to previous work cited above, we found a strong expression of nuclear phospho Ser727 STAT3 in MCL and MM as compared to normal lymphoid tissue or MGUS (see Tables 1 and 2 for the clinico-biological features). Moreover, in supplemental Figure S4 double staining of nuclear phospho Ser727 STAT3 and surface CD138 is also shown in normal (Figure S4 A), MGUS (Figure S4 B) and MM (Figure S4 C) bone marrow plasma cells. Also for this latter immunohistochemistry, the distribution of the scores of nuclear phospho Ser727 STAT3 across MGUS and MM samples is summarized in Table 3. Interestingly, 60% of the CD138+ MGUS plasma cells displayed a low staining score (0–1) for nuclear phospho Ser 727 STAT3, while 76% of CD138+ MM plasma cells displayed higher scores (2–3). Thus, in MCL and MM, similarly to phospho Ser529 NF-κB p65 (Figure S3), another CK2 target, i.e. phospho Ser727 STAT3, is highly expressed. Hence, we tested the effects of CK2 inhibitors, bortezomib and the combination of the two drugs, on STAT3 and NF-κB phosphorylation in MM and MCL cells. In MM cells, CK2 inhibition caused a strong down regulation of phospho Ser727 STAT3, both in freshly isolated malignant plasma cells from MM patients (Figure 5E) and in U-266 cells or INA-6 cells grown alone or on HS-5 stromal cells (Figure 5F, G). Also, in U-266 cells the bortezomib-induced up regulation of phospho Ser529 p65 was markedly down regulated by the combination with CK2 inhibitors (Figure 5F, G). In MCL cells, these experiments revealed comparable, though more variable, effects of CK2 inhibitors on these pro-survival signaling pathways, since CX-4945 and K27 caused a marked reduction of phospho Ser727 STAT3 and phospho Ser529 p65 in all the three MCL cell lines, which appeared milder in Granta-519 cells (Figure 5H). Altogether, these results clearly suggest that CK2 controls the extent of NF-κB and STAT3 activation in MM and MCL cells.

### CK2 down regulation by RNA interference causes higher rate of bortezomib induced MM cell apoptosis

**Figure 6:**
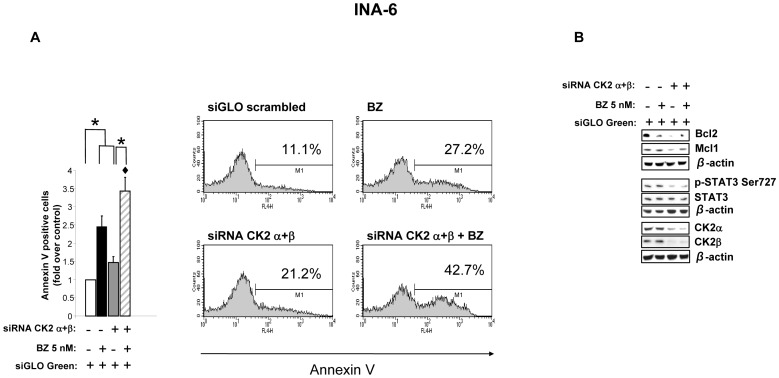
RNA interference of CK2 in MM INA-6 cells modulates apoptosis and STAT3 phosphorylation. (A) INA-6 cells were transfected with siGLO Green scrambled or siGLO Green plus CK2α and CK2βsiRNA oligos. 48h after transfection bortezomib 5 nM (BZ) was added to the cultures for 18h. Cells were collected 72h post-transfection. Apoptosis was evaluated through annexin V staining and FACS analysis on siGLO Green positive cells (A) and WB determination of the antiapoptotic markers Mcl1 and Bcl2 (B). In (A) histogram bars represent mean ± SEM of four independent experiments (left panel), while the cytometric histograms show the percentage of annexin V positive cells in a representative experiment (right panel). * indicates p<0.05. ♦ indicates p<0.05 between samples treated with bortezomib 5 nM alone and bortezomib 5 nM together with CK2 α.β siRNA. (B) STAT3 phosphorylation on Ser 727 (p-STAT3 Ser727, total STAT3, CK2α and CK2β levels were determined by WB in INA-6 tranfected with siGLO Green scrambled or siGLO Green plus CK2α and CK2βsiRNA oligos and treated with BZ for 18h. β-actin was used as a loading control.

To strengthen the findings obtained with the chemical inhibitors, we also performed RNA interference experiments against CK2α and CK2β in INA-6 cells. Cells were transfected with siGLO Green scrambled non-specific siRNAs or siGLO Green plus CK2α and CK2β-directed siRNAs and were left untreated or were treated with bortezomib (5 nM) for 18 hours. Cellular apoptosis evaluated by annexin V staining and FACS analysis demonstrated that, while both bortezomib and CK2 silencing caused a moderate amount of apoptosis, the combined treatment was followed by almost 1.5 to two-fold increase of the apoptotic rate compared to the single treatments (Figure 6A). Immunoblot analysis also demonstrated that CK2 silencing caused a reduction of Bcl2 and Mcl1 protein levels and of STAT3 phosphorylation; the levels of CK2α and CK2β were significantly reduced (Figure 6B). Thus, CK2 silencing causes INA-6 MM cell apoptosis and augments bortezomib-dependent cytotoxicity.

### CK2 inhibitors suppress NF-κB/STAT3 target gene expression in MM cells

**Figure 7:**
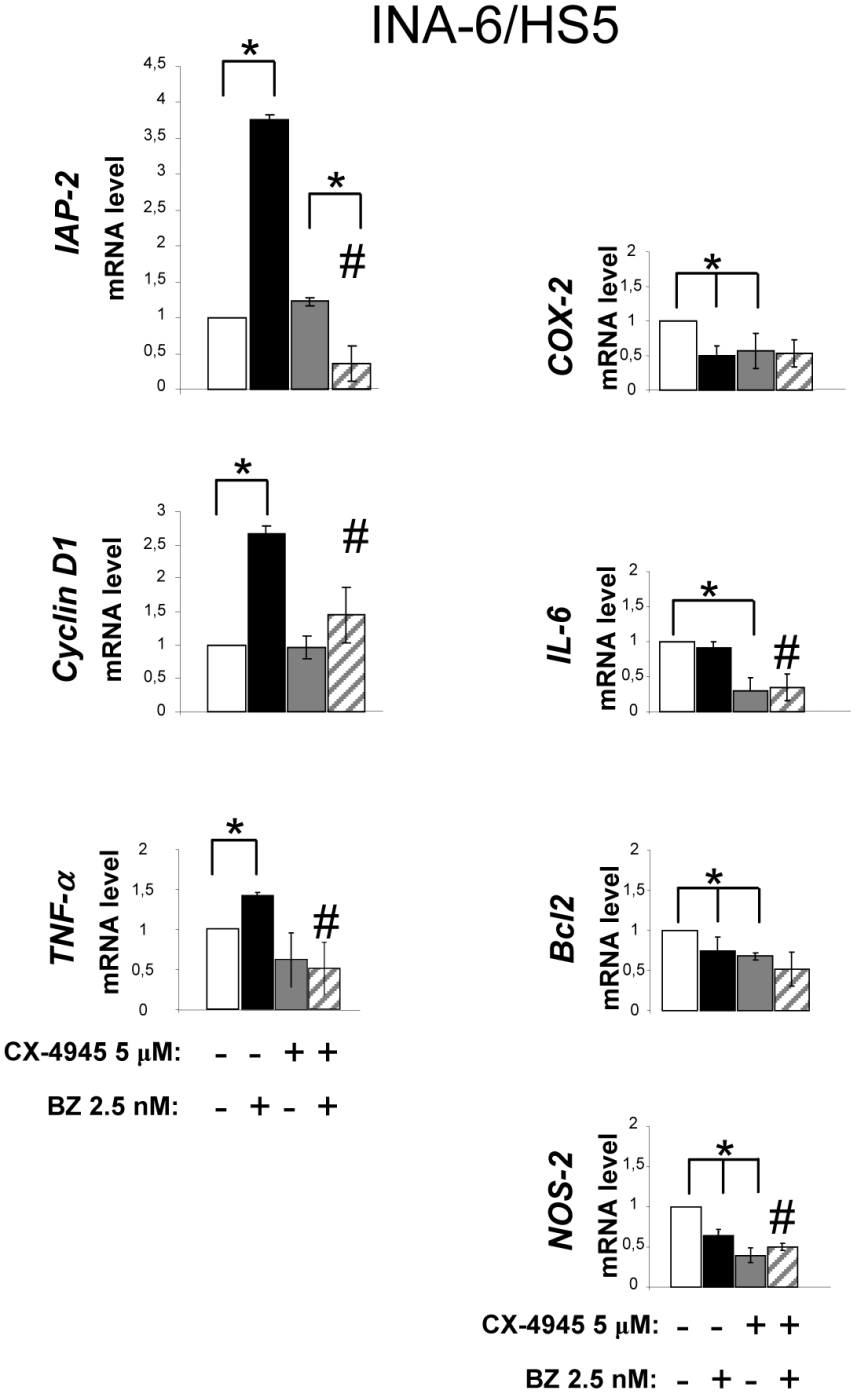
Bortezomib-induced NF-κB and STAT3 dependent genes expression is modulated by CK2. Quantitative Real Time PCR analysis of NF-**κ**B *(IAP-2, TNFα, COX-2, IL-6, Bcl2, NOS-2*) and STAT3 (*Cyclin D1, IL-6)* target gene expression performed on INA-6/HS-5 co-coltures treated with sub-apoptotic doses of bortezomib (BZ in the figure) (2.5 nM, dark bars), CX-4945 5 µM (grey bars) or the combination of the two compounds (grey striped bars) for 18 hours. Data represent mean ± SD of at least 3 independent experiments. * indicates p<0.05. # indicates p<0.05 between samples treated with bortezomib alone and bortezomib together with CX-4945.

We next assessed the expression of some pro-survival, pro-apoptotic NF-κB/STAT3 regulated genes in MM. To reproduce the MM *milieu*, we performed these experiments on INA-6 cells grown in presence of HS-5 stromal cells. Cells were treated with bortezomib, CX-4945 or with the combination of the two drugs. Quantitative real time RT-PCR was performed to analyze the mRNA levels of NF-κB *(IAP-2, TNFα, COX-2, IL-6, BCL2, NOS-2*) and STAT3 (*Cyclin D1, IL-6)* target gene expression. As shown in Figure 7, bortezomib caused an up regulation of *IAP-2, Cyclin D1 and TNFα* mRNAs that was markedly opposed by the addition of CX-4945. Furthermore, bortezomib induced a reduction of the mRNA level of the NF-κB targets *COX-2*, *BCL2* and *NOS-2*. CX-4945 strongly down regulated the expression of *COX-2, IL-6, BCL2* and *NOS-2*. These data indicate that the addition of CK2 inhibitors to bortezomib causes a block of bortezomib-induced up-regulation of some and keeps markedly down regulated other NF-κB and STAT3 targets.

### Combined treatment with bortezomib and CK2 inhibitors enhances the proteotoxic stress in MM and MCL cells

**Figure 8:**
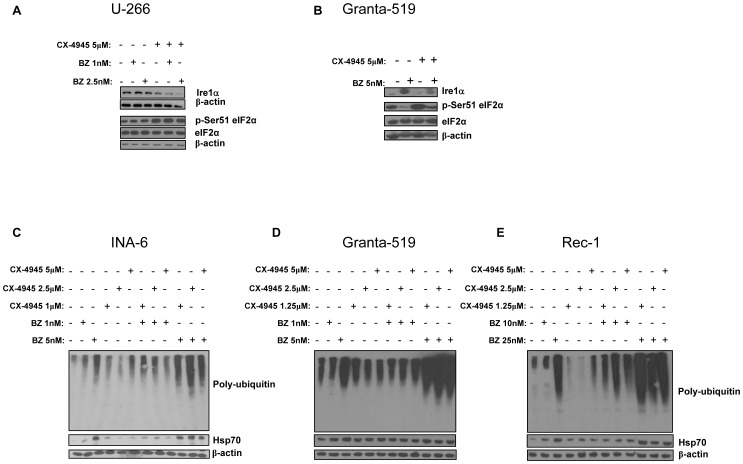
Effects of CK2 inhibition on bortezomib-induced proteotoxic/ER stress. (A, B) Expression of Ire1α, phosphorylated Ser51 eIF2α (p-Ser51 eIF2α), total eIF2α in U-266 (A) and Granta-519 (B) cells treated with the CX-4945 and bortezomib (BZ in the figure) for 6h (A) and 18h (B). (C-E) Expression of poly-ubiquitin and Hsp70 in INA-6 (C), Granta-519 (D) and Rec-1 (E) cells treated with subtoxic and toxic doses of bortezomib for 18h and subsequently incubated with bortezomib and CX-4945 at different concentrations for additional 9 hours. βactin was used as a loading control.

Since CK2 promotes a protective ER stress/UPR by maintaining the cellular levels of Ire1α [29] and facilitates the clearance of poly-ubiquitylated (poly-Ub) proteins through the autophagic cascade by mean of its phosphorylation of the docking protein p62 [49], we hypothesized that CK2 could influence the cell survival outcome upon bortezomib-induced proteotoxic stress [50]. To check if bortezomib and CK2 inhibitors could interact in the ER stress pathways and proteotoxicity, we performed WB analysis of Ire1α, total and phospho Ser51 eIF2α and poly-Ub proteins in MM cells upon exposure to bortezomib, CK2 inhibitors or the combination of the two drugs. As shown in Figure 8A,B bortezomib determined a rise of Ire1α in U-266 and Granta-519 cells and a reduction of phospho Ser51 eIF2α in Granta-519. Remarkably, the exposure to CX-4945 led to a down modulation of these effects causing a reduction of Ire1α and an increase of phospho Ser51 eIF2αprotein levels. Moreover, bortezomib led to an accumulation of poly-Ub proteins in INA-6, Granta-519 and Rec-1 cells (Figure 8 C, D, E), as shown by the appearance of a smeared signal upon probing with an anti-Ub antibody and caused an up regulation of the proteotoxic stress marker Hsp70. Interestingly, CK2 inhibition caused a mild increase of poly-Ub proteins as well, however, most remarkably, the accumulation of poly-Ub proteins was much higher in the samples treated with the combination of bortezomib and CK2 inhibitors. The detection of a parallel rise of Hsp70 protein levels - which reflects the extent of cellular proteotoxic stress - also confirmed the stronger action caused by the association of bortezomib plus CK2 inhibitors.

Thus, CK2 inhibitors act by opposing bortezomib-induced UPR changes, which could represent homeostatic compensatory mechanisms, and greatly enhance the intracellular proteotoxicity.

## Discussion

We showed here that protein kinase CK2 is highly expressed in MM and MCL and its inhibition enhances MM and MCL cells sensitivity to the proteasome inhibitor bortezomib. This effect is coupled with the down modulation of signaling pathways that may intersect with the mechanism of action of bortezomib. Our data provide the rationale for the use of CK2 inhibitors in the treatment of these malignancies.

Previously, we demonstrated a role for this kinase in MM cell survival [29], [31]. In the present work, we showed that CK2 is over expressed also in MCL and that MCL cells are induced to apoptosis when CK2 is inhibited with small ATP-competitive compounds. Our immunohistochemical analysis is, to our knowledge, the first ever reported of CK2α and CK2β expression in normal lymphoid tissue and in a subset of human non-Hodgkin lymphomas. Intriguingly, this kinase was found strongly expressed in centroblasts and centrocytes, suggesting that it could play a role in the germinal center (GC) reaction. To note, the finding of low expression levels of CK2 in the normal mantle zone, as opposed to high expression in malignant MCL cells, strongly supports a role for CK2 in the biology of MCL. We are now extending these results in MCL to GC-derived non-Hodgkin lymphomas.

We showed that CK2 dependent phosphorylation of Cdc37 and p65 NF-κB (a mirror for CK2 activity) was inducible by bortezomib and was already high in cells, which are less sensitive to bortezomib (e.g. Rec-1, Figure S3). Therefore from a clinical standpoint, it can be speculated that CK2 over expression could represent an obstacle to a fully effective cytotoxic action of this drug.

To note, CK2 modulates MCL and MM cell survival upon proteasome inhibition: MM and MCL cells undergoing treatment with CK2 inhibitors plus bortezomib were induced to a proliferation arrest that was significantly higher than what seen in cells treated with the single agents. This effect was due to a strong synergy between the two agents, as shown by the determination of the combination indexes (Figure 2). These findings confirm the efficacy of CK2 inhibitors as enhancers of other drugs' cytotoxicity, similarly to what we recently demonstrated using the CK2 inhibitor tTBB in association with the Hsp90 inhibitor 17-AAG. This last approach potently inhibited MM cell growth in a mouse xenograft model and provided the first *in vivo* evidence of an anti-myeloma effect of CK2 targeting [29]. The cooperation between CK2 inhibitors and bortezomib was confirmed in different experimental settings, including a MM microenvironment model using the stromal cells HS-5 and freshly isolated cells from MM patients (Figure 3 and S2). Most importantly, RNA interference and silencing of the kinase with siRNAs directed against CK2α and CK2β reproduced the effects obtained with the chemical inhibitors, confirming a cooperation with bortezomib in inducing cell apoptosis of INA-6 MM cells (Figure 6A). Significantly, normal B lymphocytes, were fairly less sensitive to CK2 and proteasome inhibition (Figure S2C). These findings were substantiated by numerous cell viability assays and evaluation of the homeostatic mitochondrial mechanism (Figure 4 and S2D). Notably, the cooperation between bortezomib and CK2 inhibitors was effective also using doses of bortezomib that are sub-apoptotic when added alone, both in U-266 MM cells and also in MCL Rec-1 cells, which are less sensitive to bortezomib [40]. Overall, these data clearly indicate that CK2 activity could somewhat lie downstream from bortezomib and that this protein kinase could partly counteract bortezomib-induced apoptosis. Also, they suggest that the less sensitive the cells to bortezomib, the higher the levels and activity of CK2 and its targets. Indeed, high levels of phosphorylated Ser529 NF-κB p65 and Ser727 STAT3 paralleled the strong expression of CK2 in MCL and MM was (Figures S3, S4 and 5). Most importantly, CK2 inhibition obtained with chemicals caused a consistent drop of the levels of phospho NF-κB and phospho STAT3 in MM and MCL cells, with a consequent down-regulation of a set of target genes. Also, CK2-directed RNA interference experiments confirmed, in INA-6 cells, that CK2 sustains the phosphorylation levels of STAT3 (Figure 6B).

These findings are consistent with previous descriptions suggesting that CK2 might control NF-κB and STAT3 activation in MM cells and likely in other tumors [22], [31]. Since bortezomib can also trigger the activation of the NF-κB pathway [10], our results are particularly meaningful in the perspective of developing strategies limiting NF-κB activation upon proteasome inhibition. Similarly, STAT3 downmodulation upon CK2 inhibition could represent an exploitable therapeutic strategy. CK2 can regulate STATs signaling by impinging on JAKs activation and by direct phosphorylation of STATs, as demonstrated for STAT1 and STAT3 [51], [52], [53], [54]. It will be important to elucidate the exact mechanism of CK2 action on these two cascades in MM and MCL cells.

We also provided evidence for another level of regulation of the bortezomib-induced cell response by CK2, namely the ER stress/UPR and the proteotoxic stress. Ire1α, the ER-localized sensor, is responsible for a compensatory response upon ER stress. We recently demonstrated that the levels and activity of Ire1α are influenced by CK2 [29]. Our findings emphasize the importance of this kinase in sustaining a pro-survival response not only upon ER stress or Hsp90 inhibition but also upon bortezomib treatment. Interestingly, phospho Ser51 eIF2α levels were markedly increased upon CK2 inhibition in MM and MCL cells, irrespective of the effects of bortezomib on this branch of the UPR. Since it has been proposed that maintaining high the levels of phospo Ser51 eIF2α could potentiate the cytotoxic mechanism of bortezomib [55], our result suggest the use of CK2 inhibitors to achieve this goal. As a consequence of an increased uncompensated UPR, we could demonstrate that CK2 inhibition enhanced the accumulation of poly-Ub proteins and of Hsp70 upon proteasome block (Figure 8C, D, E). Therefore, CK2 could control the proteotoxic stress response in MM cells.

In conclusion, in this work we provide evidence that CK2 inhibition could represent a powerful way to boost bortezomib-mediated cell death of MM and MCL. CK2 inhibitors may simultaneously down modulate critical pro-survival pathways, producing a multisided "wounding effect" on bortezomib-treated malignant B cells. It is conceivable that the use of CK2 inhibitors might minimize the likelihood of the emergence of bortezomib-resistant clones and could improve the overall response to this and other second-generation proteasome inhibitors.

## Supporting Information

Figure S1 **Double immunohistochemical staining analysis of CD138 and CK2α, CK2β in normal, MGUS and MM BM biopsies.** Plasma cell specific marker CD138 staining is shown in red and CK2α or CK2β are shown in brown in representative normal bone marrow (A, B), MGUS (C, D) and MM samples (E, F). Immunoperoxidase stain, original magnification 20x.
(PPT)

Figure S2 **Effects of CK2 inhibitors and bortezomib on MM and MCL survival in different experimental conditions.** (A) Quantification of apoptosis through annexin V staining and FACS analysis in MM cells INA-6 alone (leftmost panel), or in INA-6 grown in co-cultures with the human bone marrow stroma cell line HS-5 (rightmost panel), treated with doxorubicin 1.2 µM for 18h. (B-C) Quantification of apoptosis through annexin V staining and FACS analysis (top panel) or WB analysis of PARP cleavage (bottom panel) in MM cells U-266 (B, leftmost panel), INA-6 (B, middle panel), INA-6 co-cultures grown with the human bone marrow stroma cell line HS-5 (B, rightmost panel), normal B lymphocytes (C) treated with K27 (dark grey bar) or CX-4945 (light grey bars), bortezomib (BZ in the figure) at different concentrations (black bars) or the combination of K27 or CX-4945 and bortezomib (grey striped bars for K27 together with BZ or grey dotted bars for CX-4945 together with BZ) for 18h. In the case of INA-6 grown in co-colture with HS-5 experiments were performed by staining with APC-conjugated anti-CD45 antibody, which is expressed by INA-6 cells but not by stromal cells and with FITC-conjugated annexin V. * indicates p<0.05. In B # indicates p<0.05 between samples treated with bortezomib 1 nM alone and bortezomib 1 nM together with K27. ♦ indicates p<0.05 between samples treated with bortezomib 5 nM alone and bortezomib 5 nM together with K27. (D) ATP measurement in MM (INA-6, leftmost panel) or MCL (Rec-1, rightmost panel) treated with K27 or CX-4945 and bortezomib at the doses indicated in figure. * indicates p<0.05. # indicates p<0.05 between samples treated with bortezomib alone and bortezomib together with K27 or CX-4945. In the entire figure data are presented as mean ± SEM and are representative of at least 3 independent experiments.
(PPT)

Figure S3 **Bortezomib induces CK2 activation in MM and MCL cell lines.** WB analysis of CK2 target phospho-proteins (phosho Cdc37 Ser13, phospho NF-κB p65 Ser529) and their total forms in MM or MCL cell lines treated with bortezomib (BZ in the figure) for 8h at the concentrations indicated in figure. β actin was used as a loading control.
(PPT)

Figure S4 **Double immunohistochemical staining analysis of CD138, phospho Ser727 STAT3 in normal, MGUS and MM BM biopsies.** Plasma cell specific marker CD138 staining is shown in red and phospho STAT3 Ser727 is shown in brown in representative normal bone marrow (A), MGUS (B) and MM samples (C). Original magnification 20x.
(PPT)
