# Supplementary figures and images for: Protein Kinase CK2 Inhibition Down Modulates the NF-κB and STAT3 Survival Pathways, Enhances the Cellular Proteotoxic Stress and Synergistically Boosts the Cytotoxic Effect of Bortezomib on Multiple Myeloma and Mantle Cell Lymphoma Cells
Source: PLoS One. 2013 Sep 27;8(9):e75280. doi: 10.1371/journal.pone.0075280 (PMC3785505; doi:10.1371/journal.pone.0075280)

## Slide 1
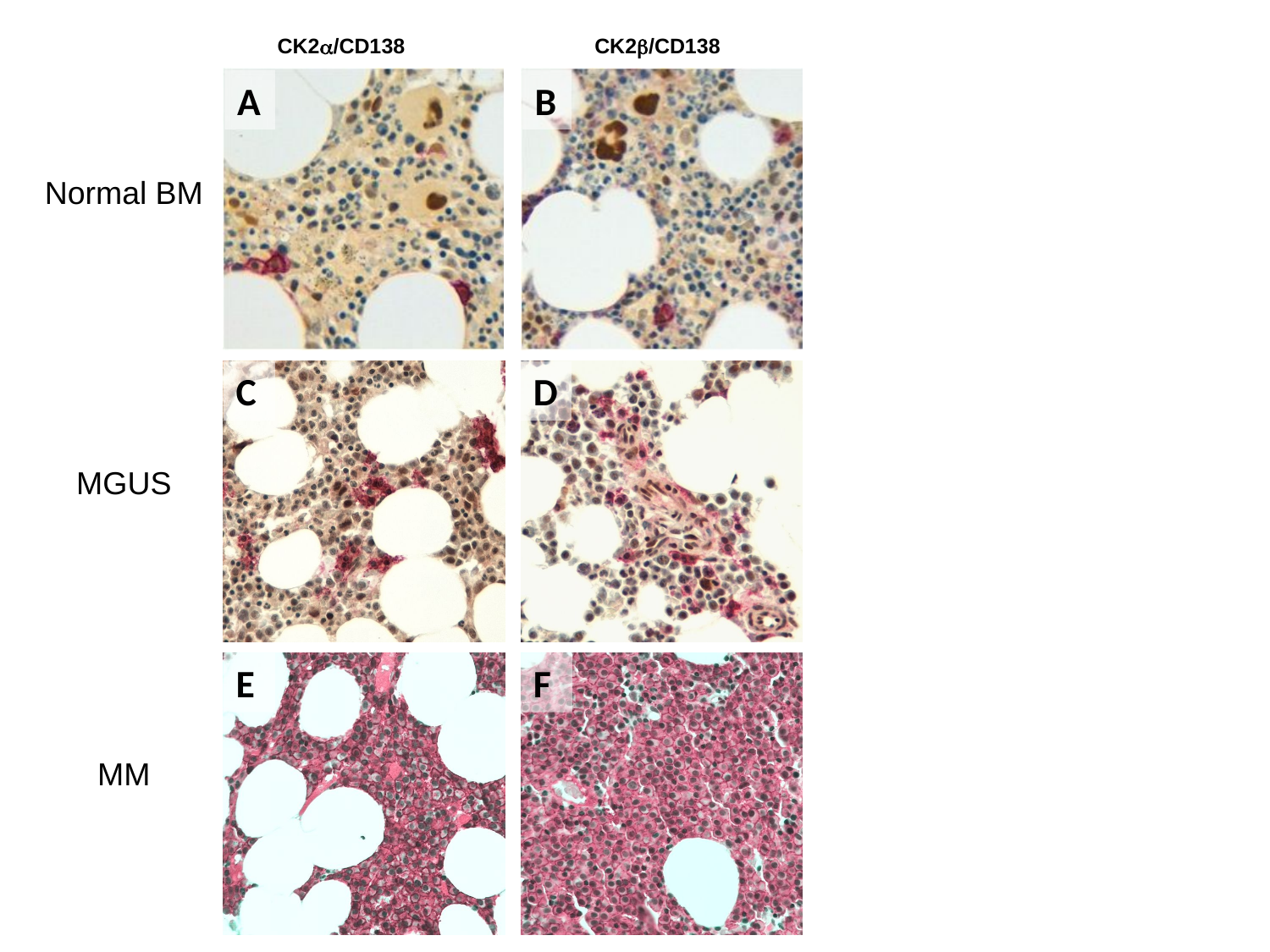

CK2α/CD138
CK2β/CD138
A
B
Normal BM
C
D
MGUS
E
F
MM

Supplement: Figure S1 — Double immunohistochemical staining analysis of CD138 and CK2α, CK2β in normal, MGUS and MM BM biopsies. Plasma cell specific marker CD138 staining is shown in red and CK2α or CK2β are shown in brown in representative normal bone marrow (A, B), MGUS (C, D) and MM samples (E, F). Immunoperoxidase stain, original magnification 20x. (PPT) [file pone.0075280.s001.ppt]

## Slide 1
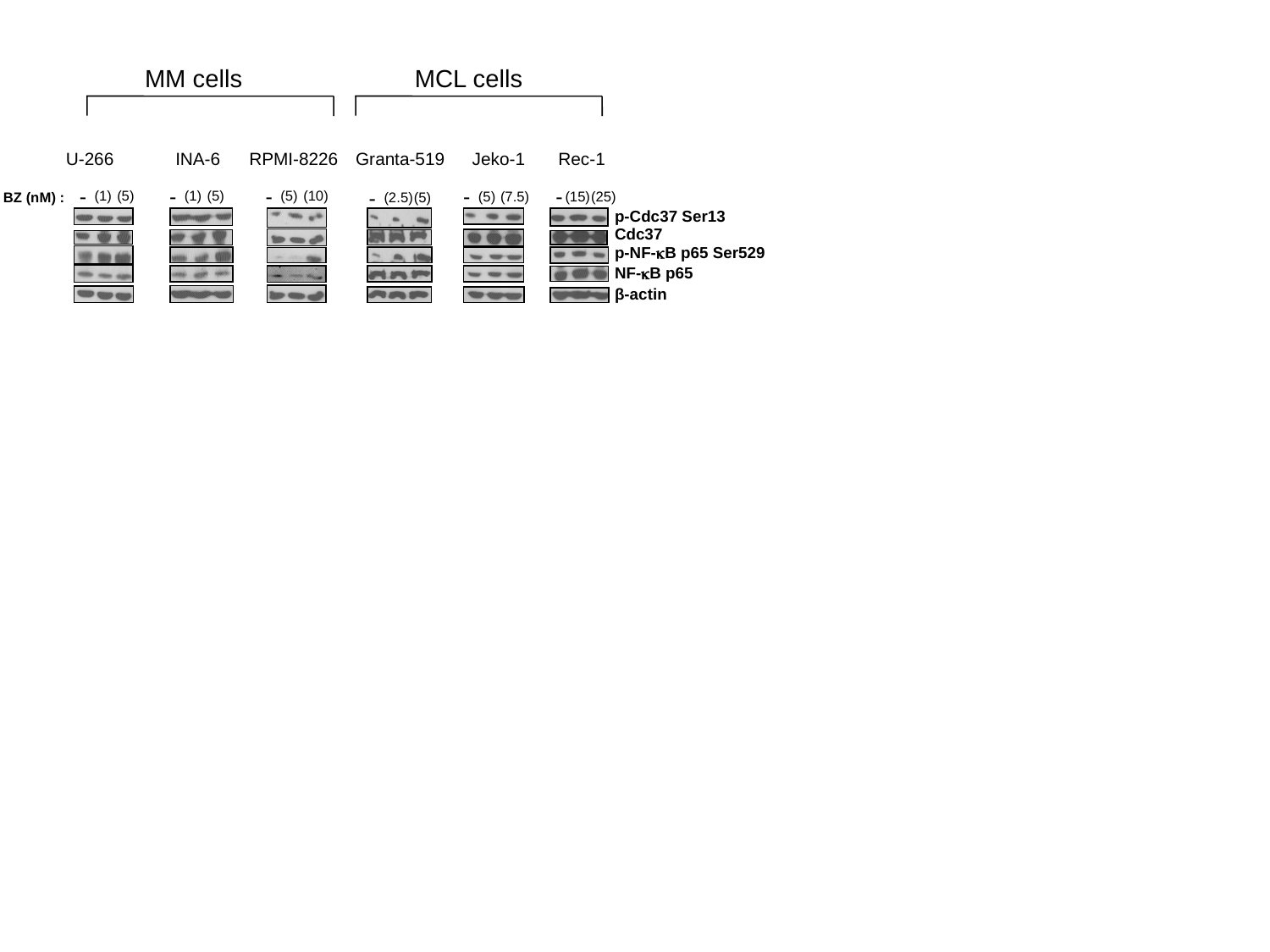

MM cells
MCL cells
U-266
INA-6
RPMI-8226
Granta-519
Jeko-1
Rec-1
-
(1)
(5)
-
(1)
(5)
-
(5)
(10)
BZ (nM) :
-
(5)
(7.5)
-
(15)
(25)
-
(2.5)
(5)
p-Cdc37 Ser13
Cdc37
p-NF-κB p65 Ser529
NF-κB p65
β-actin

Supplement: Figure S3 — Bortezomib induces CK2 activation in MM and MCL cell lines. WB analysis of CK2 target phospho-proteins (phosho Cdc37 Ser13, phospho NF-κB p65 Ser529) and their total forms in MM or MCL cell lines treated with bortezomib (BZ in the figure) for 8h at the concentrations indicated in figure. β actin was used as a loading control. (PPT) [file pone.0075280.s003.ppt]

## Slide 1
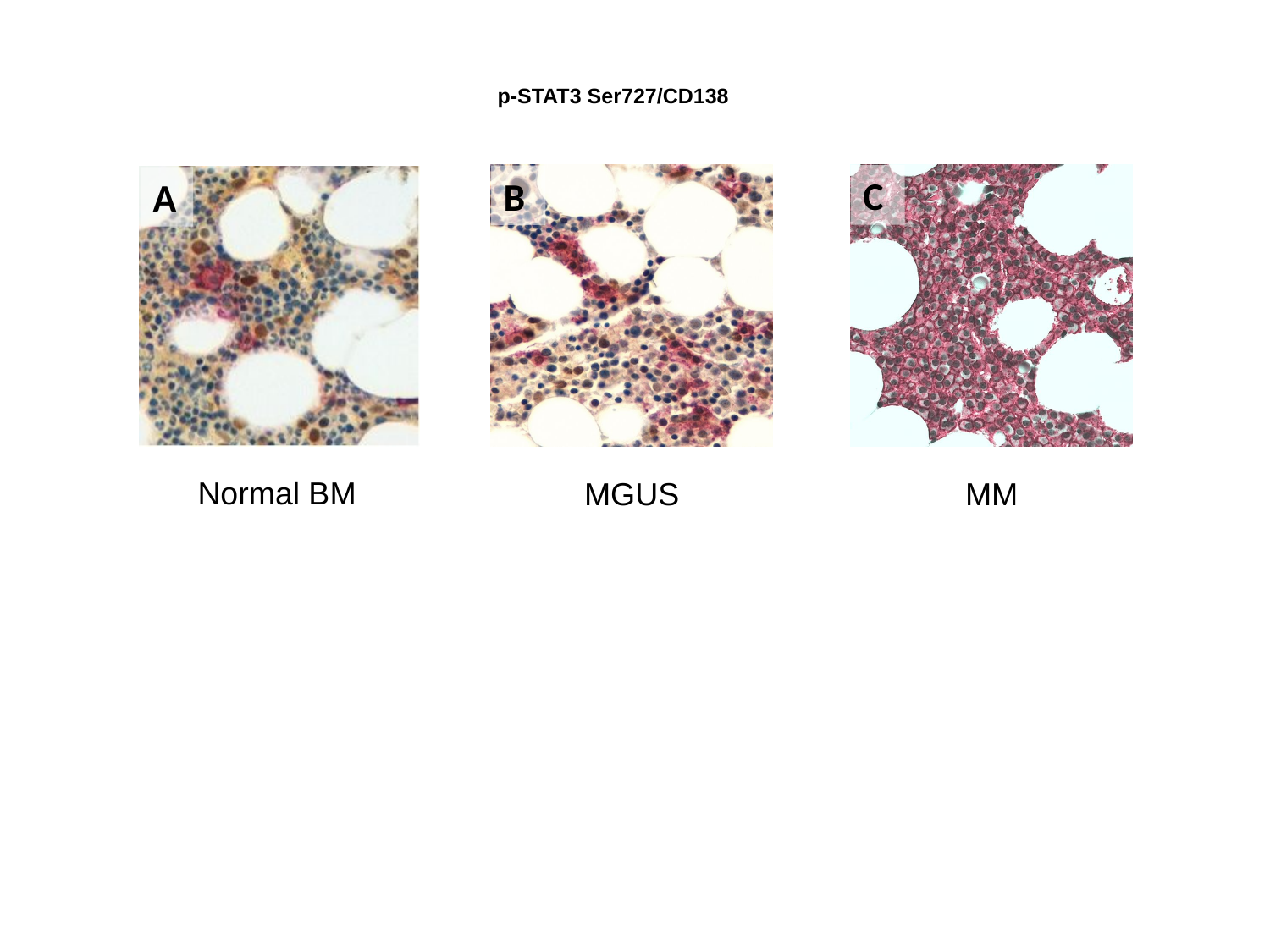

p-STAT3 Ser727/CD138
B
C
A
Normal BM
MGUS
MM

Supplement: Figure S4 — Double immunohistochemical staining analysis of CD138, phospho Ser727 STAT3 in normal, MGUS and MM BM biopsies. Plasma cell specific marker CD138 staining is shown in red and phospho STAT3 Ser727 is shown in brown in representative normal bone marrow (A), MGUS (B) and MM samples (C). Original magnification 20x. (PPT) [file pone.0075280.s004.ppt]
