# Supplementary material for: Protein Kinase CK2 Inhibition Down Modulates the NF-κB and STAT3 Survival Pathways, Enhances the Cellular Proteotoxic Stress and Synergistically Boosts the Cytotoxic Effect of Bortezomib on Multiple Myeloma and Mantle Cell Lymphoma Cells
Source: PLoS One. 2013 Sep 27;8(9):e75280. doi: 10.1371/journal.pone.0075280 (PMC3785505; doi:10.1371/journal.pone.0075280)
Supplement: Figure S2 — Effects of CK2 inhibitors and bortezomib on MM and MCL survival in different experimental conditions. (A) Quantification of apoptosis through annexin V staining and FACS analysis in MM cells INA-6 alone (leftmost panel), or in INA-6 grown in co-cultures with the human bone marrow stroma cell line HS-5 (rightmost panel), treated with doxorubicin 1.2 µM for 18h. (B-C) Quantification of apoptosis through annexin V staining and FACS analysis (top panel) or WB analysis of PARP cleavage (bottom panel) in MM cells U-266 (B, leftmost panel), INA-6 (B, middle panel), INA-6 co-cultures grown with the human bone marrow stroma cell line HS-5 (B, rightmost panel), normal B lymphocytes (C) treated with K27 (dark grey bar) or CX-4945 (light grey bars), bortezomib (BZ in the figure) at different concentrations (black bars) or the combination of K27 or CX-4945 and bortezomib (grey striped bars for K27 together with BZ or grey dotted bars for CX-4945 together with BZ) for 18h. In the case of INA-6 grown in co-colture with HS-5 experiments were performed by staining with APC-conjugated anti-CD45 antibody, which is expressed by INA-6 cells but not by stromal cells and with FITC-conjugated annexin V. * indicates p<0.05. In B # indicates p<0.05 between samples treated with bortezomib 1 nM alone and bortezomib 1 nM together with K27. ♦ indicates p<0.05 between samples treated with bortezomib 5 nM alone and bortezomib 5 nM together with K27. (D) ATP measurement in MM (INA-6, leftmost panel) or MCL (Rec-1, rightmost panel) treated with K27 or CX-4945 and bortezomib at the doses indicated in figure. * indicates p<0.05. # indicates p<0.05 between samples treated with bortezomib alone and bortezomib together with K27 or CX-4945. In the entire figure data are presented as mean ± SEM and are representative of at least 3 independent experiments. (PPT) [file pone.0075280.s002.ppt]

## Slide 1
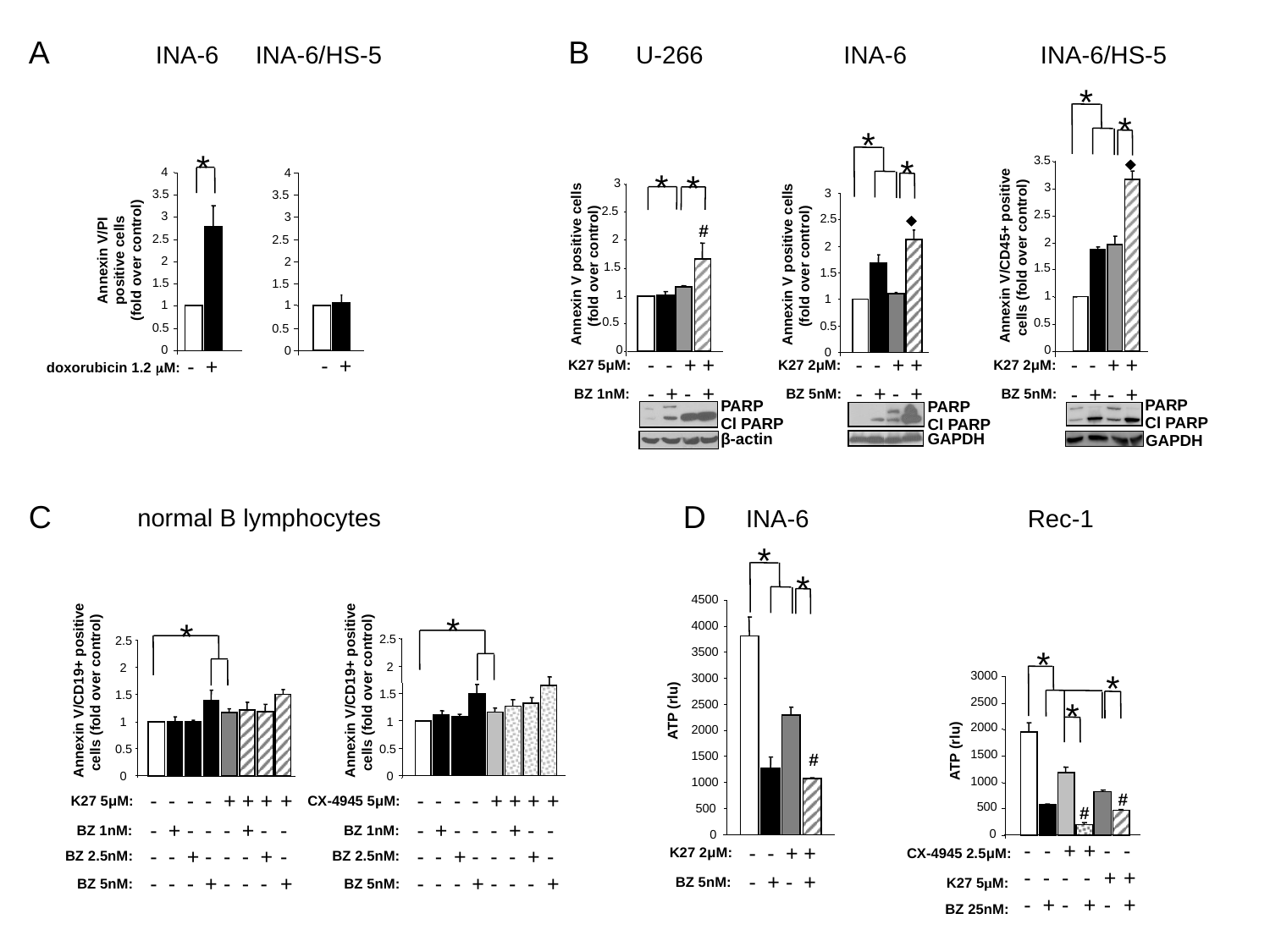

A
INA-6
INA-6/HS-5
*
4
3.5
3
2.5
2
1.5
1
0.5
0
4
3.5
3
2.5
2
1.5
1
0.5
0
-
+
Annexin V/PI positive cells (fold over control)
-
+
doxorubicin 1.2 μM:
B
U-266
INA-6
INA-6/HS-5
*
*

3.5
3
2.5
2
1.5
1
0.5
0
Annexin V/CD45+ positive cells (fold over control)
*
*
*
*
3
3
2.5
2
1.5
1
0.5
0

2.5
#
2
Annexin V positive cells (fold over control)
Annexin V positive cells (fold over control)
1.5
1
0.5
0
-
-
+
+
K27 5μM:
-
+
-
+
BZ 1nM:
-
-
+
+
K27 2μM:
-
+
-
+
BZ 5nM:
-
-
+
+
K27 2μM:
-
+
-
+
BZ 5nM:
PARP
Cl PARP
PARP
Cl PARP
PARP
Cl PARP
GAPDH
β-actin
GAPDH
C
normal B lymphocytes
*
2.5
2
1.5
1
0.5
0
Annexin V/CD19+ positive cells (fold over control)
-
-
-
-
+
+
+
+
-
+
-
-
-
+
-
-
BZ 1nM:
-
-
+
-
-
-
+
-
BZ 2.5nM:
-
-
-
+
-
-
-
+
BZ 5nM:
CX-4945 5μM:
*
2.5
2
1.5
1
0.5
0
Annexin V/CD19+ positive cells (fold over control)
-
-
-
-
+
+
+
+
K27 5μM:
-
+
-
-
-
+
-
-
BZ 1nM:
-
-
+
-
-
-
+
-
BZ 2.5nM:
-
-
-
+
-
-
-
+
BZ 5nM:
D
INA-6
*
*
4500
4000
3500
3000
2500
2000
1500
1000
500
0
ATP (rlu)
#
-
-
+
+
K27 2μM:
-
+
-
+
BZ 5nM:
*
*
3000
2500
2000
1500
1000
500
0
*
ATP (rlu)
#
#
-
-
+
+
-
-
-
-
-
-
+
+
+
-
+
-
+
-
CX-4945 2.5μM:
K27 5μM:
BZ 25nM:
Rec-1
